# Clinical Prognostic Impact of CALLY in Heart Failure Patients With Different Ejection Fractions: A Retrospective Study

**DOI:** 10.31083/RCM49108

**Published:** 2026-08-25

**Authors:** Xinuo Ma, Jianping Yang, Zijie Liu, Ying Li, Sirui Yang, Tao Shi, Fazhi Yang, Yujuan Peng, Lixing Chen

**Affiliations:** ^1^Department of Cardiology, The First Affiliated Hospital of Kunming Medical University, 650032 Kunming, Yunnan, China; ^2^College of Big Data, Yunnan Agricultural University, 650201 Kunming, Yunnan, China; ^3^The Key Laboratory for Crop Production and Smart Agriculture of Yunnan Province, 650201 Kunming, Yunnan, China; ^4^Department of Clinical Laboratory, The First Affiliated Hospital of Kunming Medical University, 650032 Kunming, Yunnan, China; ^5^Internal Medicine Department for Officials, The First Affiliated Hospital of Kunming Medical University, 650032 Kunming, Yunnan, China

**Keywords:** heart failure, inflammation, nutrition, prognosis, ejection fraction

## Abstract

**Background:**

Chronic heart failure (CHF) is closely linked to high morbidity and mortality rates, with inflammation and changes in nutritional status being key contributors to disease progression. Thus, this study aimed to evaluate the prognostic utility of the novel composite index CALLY, which incorporates albumin (ALB) level, lymphocyte count (LYM), and C-reactive protein (CRP) level, for predicting all-cause mortality in patients with CHF and varying ejection fractions.

**Methods:**

This retrospective cohort study included 1193 patients with CHF who were admitted to the First Affiliated Hospital of Kunming Medical University between January 2017 and October 2021. Restricted cubic spline analysis was used to determine the CALLY index cutoff value and classify patients into two groups. Kaplan–Meier and multivariable Cox regression analyses were performed to assess the prognostic value of CALLY in the overall cohort and in subgroups with HFrEF, HFmrEF, and HFpEF. Model performance was evaluated using receiver operating characteristic curves with DeLong’s test, calibration curves with bootstrap resampling, and decision curve analysis.

**Results:**

Kaplan‒Meier analysis revealed that lower CALLY levels were associated with higher all-cause mortality in all patients with CHF and in the twoCHF subgroups. Multivariable Cox proportional hazards analysis demonstrated that CALLY independently predicted all-cause mortality across all heart failure subtypes. Receiver operating characteristic curve analysis indicated that models incorporating CALLY exhibited improved predictive performance. Calibration and decision curve analyses further supported the clinical utility of the CALLY index.

**Conclusions:**

Amongpatients with CHF in this cohort, a lower CALLY index was independently associated with higher all-cause mortality across different ejection fraction categories. These findings suggest that CALLY may serve as a potential risk marker.

## 1. Introduction

Heart failure (HF) is the final stage of various cardiovascular diseases and has become a major public health issue because of its poor prognosis and high rehospitalization rate. According to the Global Burden of Disease (GBD) study, the global prevalence of heart failure increased from 25.43 million cases in 1990 to 55.50 million cases in 2021, more than doubling [1]. Moreover, as the largest developing country, China also experienced a significant increase in its incidence rate, which is approximately 208.4% greater than that of 30 years ago, reaching 14.3 million cases by 2023 [2]. Therefore, quickly identifying and diagnosing high-risk populations for heart failure is crucial.

The 2021 European Society of Cardiology Heart Failure Guidelines classify HF into three phenotypes on the basis of on left ventricular ejection fraction (LVEF): heart failure with reduced ejection fraction (HFrEF, LVEF ≤40%), heart failure with mildly reduced ejection fraction (HFmrEF, LVEF 41%–49%), and heart failure with preserved ejection fraction (HFpEF, LVEF ≥50%) [3].The pathogenesis of chronic heart failure (CHF) involves immune-inflammatory dysregulation and metabolic disorders, and multiple interrelated mechanisms lead to deterioration of nutritional status [4]. In previous studies, the systemic immune inflammation index (SII), neutrophil-to-lymphocyte ratio (NLR), platelet-to-lymphocyte ratio (PLR), prognostic nutritional index (PNI), and CONUT score alone have been shown to solely explain the effects of inflammation or immune mechanisms on heart failure [5,6,7].

The CALLY index, a novel inflammatory–nutritional–immune composite index, has been previously demonstrated to have predictive value for the prognosis of diseases such as gastrointestinal tumors, chronic obstructive pulmonary disease (COPD), Kawasaki disease, and stroke [8,9,10,11]. In the field of cardiovascular diseases, CALLY has been confirmed to be associated with adverse cardiovascular outcomes (MACEs) in patients with acute ST-segment elevation myocardial infarction (STEMI) [12]. However, its application in different types of heart failure has not yet been reported. The aim of this retrospective cohort study was to investigate the relationship between CALLY levels and all-cause mortality in CHF patients with different ejection fractions.

## 2. Materials and Methods

### 2.1 Study Population

This retrospective cohort study was conducted at the First Affiliated Hospital of Kunming Medical University. A total of 1221 consecutive patients with chronic heart failure (CHF) admitted between January 2017 and October 2021 were initially screened for eligibility. The inclusion criteria were NYHA functional class III/IV status and a B-type natriuretic peptide (BNP) concentration ≥500 pg/mL. The exclusion criteria were (1) incomplete medical records (e.g., missing laboratory or imaging data), (2) comorbid severe conditions (malignancy, active infections, hematologic disorders, or advanced renal/hepatic dysfunction), and (3) inadequate follow-up duration. Following rigorous screening, 1193 patients were included in the final analysis. The flow diagram of patient selection is presented in **Supplementary Fig. 1**. Due to the sequential nature of the screening process, the exact number of patients excluded for each individual criterion could not be retrospectively quantified.

### 2.2 Data Collection and Definitions

Comprehensive clinical data encompassing demographic characteristics, medical history, treatment regimens, and laboratory parameters were extracted from the patients electronic medical records. At the time of admission, no treatment measures were taken and after a 12-hour fasting period, routine hematological and biochemical parameters were collected from the elbow vein for baseline laboratory tests. Laboratory analyses included complete blood count, BNP concentration, myoglobin level, creatine kinase MB isoenzyme (CK-MB) level, cardiac troponin I (cTnI) level, electrolyte (sodium, potassium, and chloride) levels, albumin (ALB) level, uric acid (UA) level, glomerular filtration rate (GFR), and liver enzyme [alanine aminotransferase (ALT), and aspartate aminotransferase (AST)] levels, along with inflammatory markers. All the samples were processed immediately in the hospital’s central laboratory using standardized protocols. An echocardiogram was completed within 3 days after admission. If a patient is readmitted to the hospital for examination because of worsening conditions, we record the time of the first examination and the relevant data. Survival outcomes were obtained retrospectively via telephone interviews with patients or their relatives, with all-cause mortality serving as the primary endpoint.

### 2.3 Calculation of CALLY

The CALLY composite index was calculated using the formula [13]:


$$CALLY=\frac{\text{Albumin}\left(g/dL\right)\times \text{Lymphocyte Count(}\times 10^{9}/L)}{C-\text{Reactive Protein}(mg/L)}$$

### 2.4 Statistical Analysis

Normality assessment of continuous variables was performed using the Shapiro‒Wilk test. Variables were classified as normally or nonnormally distributed on the basis of the test results. Normally distributed variables are expressed as the mean ± standard deviation (SD), whereas nonnormally distributed variables are reported as the median [interquartile range (IQR)]. Categorical variables are summarized as frequencies and percentages. For comparisons between the patient groups with different CALLY levels, an independent sample *t* test was used for comparisons of normally distributed data. The Mann–Whitney U test was used for comparisons of nonnormally distributed data. The chi-square test was used for comparisons of categorical data. Scatter plots were constructed to demonstrate the correlations between the components of the CALLY index (albumin, CRP, and lymphocytes). Spearman rank correlation analysis was used to evaluate the correlation of the CALLY index with the PNI and the CONUT score, which is presented as a scatter plot matrix.

Restricted cubic spline (RCS) regression with four knots placed at the 5th, 35th, 65th, and 95th percentiles of the CALLY distribution was used to flexibly model the relationship between the CALLY index and mortality risk. The analysis revealed a significant non-linear association (*p* for non-linearity < 0.001). Given this non-linearity, traditional cutoff selection methods that assume a monotonic relationship (e.g., maximizing the Youden index) are less appropriate. Instead, the optimal cutoff value was defined as the point where the adjusted hazard ratio (HR) curve crosses HR = 1 (cutoff = 0.62), indicating that above this threshold, the mortality risk is consistently lower than the reference level. Based on this cutoff, all patients were divided into low CALLY (<0.62) and high CALLY (≥0.62) groups. To assess the robustness of our findings and address concerns about information loss due to dichotomization, sensitivity analyses were also performed using CALLY Tertiles.

Kaplan‒Meier survival curves were used to compare all-cause mortality between groups, with differences evaluated by the log-rank test. The following Cox proportional hazards regression models were constructed, and the variables for which *p* was <0.05 in the univariate analysis were included in the multivariate Cox analysis. The following groups were included in the analysis: ① the HFrEF plus HFmrEF group and HFpEF group; ② the HFmrEF subgroup; and ③ all patients with heart failure.

To evaluate the predictive value of the CALLY index, three basic models (traditional covariates) and a CALLY model (a basic model plus the CALLY index) were constructed. The ROC curve was used to compare the discrimination of the four models, the differences in AUC values were evaluated by the DeLong test, and the sensitivity and specificity of the models were reported. The calibration curve was used to evaluate the goodness of fit of the models, and the bootstrap method (200 repeated samples) was used to calculate the C-index of the CALLY model, which is presented together with the calibration curve. Decision curve analysis (DCA) was used to assess the net clinical benefit. The proportional hazards assumption of the Cox model was validated with the use of a Schoenfeld residual global test.

Statistical analyses were conducted with SPSS version 25.0 (IBM Corp., Armonk, NY, USA) and R version 4.2.0 (R Foundation for Statistical Computing, Vienna, Austria). A two-sided *p* value < 0.05 was considered to indicate statistical significance.

## 3. Results

### 3.1 Baseline Patient Characteristics

**Fig. 1.**
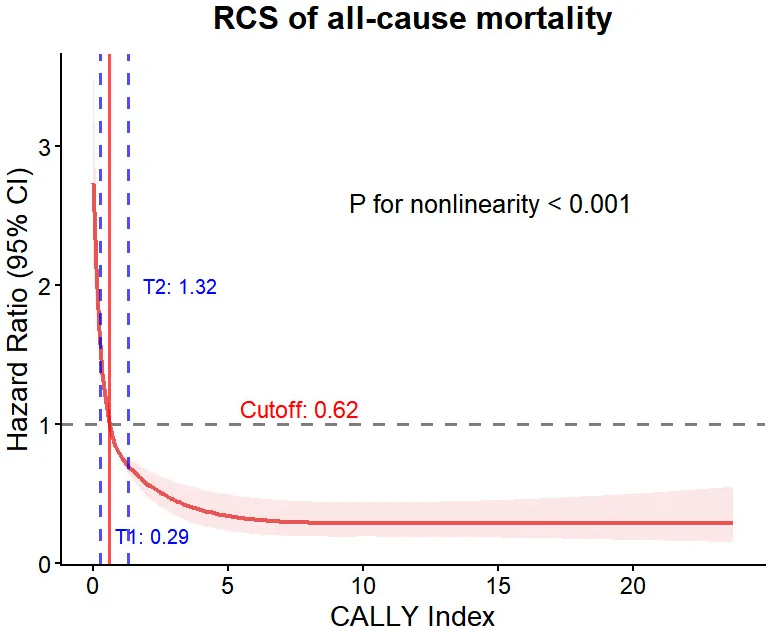
**Restricted cubic spline analysis of CALLY**.

After excluding 28 individuals, 1193 individuals diagnosed with heart failure (age: 66.86 ± 12.52 years; 38% female) were included in the final analysis. Using the CALLY optimal threshold of 0.62 (*p* < 0.001), we constructed a restricted cubic spline plot to stratify patients into two groups (Fig. 1). Baseline characteristics were compared between these groups (Table 1). Multiple clinical parameters differed significantly between the low-CALLY group (n = 598) and the high-CALLY group (n = 595) during a median follow-up period of 755 days. Compared with the low-CALLY group, the high-CALLY group exhibited a lower mean age, log-transformed B-type natriuretic peptide (lgBNP) level, neutrophil (Neu) count, NLR, C-reactive protein (CRP) level, alanine aminotransferase (ALT) level, aspartate aminotransferase (AST) level, creatinine level, uric acid (UA) level, fasting blood glucose (FBG), and total bilirubin (TB) level (*p* < 0.05). Conversely, the high-CALLY group had a higher red blood cell (RBC) count, lymphocyte (LYM) count, hemoglobin (Hb) level, platelet (PLT) count, sodium concentration, chloride concentration, albumin (ALB) level, total cholesterol (TC) level, glomerular filtration rate (GFR), high-density lipoprotein cholesterol (HDL-C) level, low-density lipoprotein cholesterol (LDL-C level), and left ventricular diameter (LVd) (*p* < 0.05). With respect to medication history, the proportion of patients who used oral beta-receptor blockers and diuretics was significantly greater in the high-CALLY group. Moreover, the prevalence of comorbidities including coronary heart disease, diabetes mellitus, hypertension, atrial fibrillation, and stroke, as well as the use of sodium-dependent glucose transporter 2 inhibitors (SGLT-2i) and ACEI/ARB/ARNI medications (angiotensin-converting enzyme inhibitors/angiotensin receptor blockers/angiotensin receptor neprilysin inhibitors), was lower in the high-CALLY group (*p* < 0.05).

**Table 1.** **Baseline characteristics of the low-CALLY and high-CALLY groups**.

Characteristics		Total (n = 1193)	low-CALLY (n = 598)	high-CALLY (n = 595)	*p* value
Basic characteristics					
	Age (years)	66.86 ± 12.52	68.90 ± 11.83	64.49 ± 12.70	<0.001
	Female, n (%)	453 (38.00%)	213 (35.60%)	240 (40.30%)	0.093
	Heart Rate (bpm)	86.25 ± 20.50	86.94 ± 20.48	85.10 ± 20.38	<0.05
	SBP (mmHg)	122.15 ± 22.94	121.13 ± 22.69	123.27 ± 22.99	<0.05
	DBP (mmHg)	76.28 ± 15.02	75.17 ± 14.67	77.22 ± 15.31	<0.05
	BMI (kg/m^2^)	23.02 ± 3.82	22.91 ± 3.62	23.17 ± 3.99	0.644
	NYHA class Ⅳ, n (%)	444 (37.20%)	250 (41.80%)	194 (32.60%)	<0.001
Medical history, n (%)					
	Coronary artery disease	617 (51.70%)	317 (53.00%)	300 (50.40%)	0.371
	Hypertension	659 (55.20%)	333 (55.70%)	326 (54.80%)	0.756
	Diabetes	339 (28.40%)	189 (31.60%)	150 (25.20%)	<0.05
	Atrial fibrillation	406 (34.00%)	208 (34.80%)	198 (33.30%)	0.583
	Stroke	408 (34.20%)	213 (35.60%)	195 (32.80%)	0.300
Treatment					
	SGLT-2i	269 (22.50%)	149 (24.90%)	120 (20.20%)	<0.05
	β-receptor blockers	837 (70.2%)	414 (69.20%)	423 (71.10%)	0.482
	Diuretics	941 (78.9%)	470 (78.60%)	471 (79.20%)	0.811
	ACEI/ARB/ARNI	670 (56.2%)	340 (56.90%)	330 (55.50%)	0.628
	CRT/CRT-D	116 (9.70%)	57 (9.50%)	59 (9.90%)	0.823
Laboratory indicators					
	lgBNP	3.17 ± 0.28	3.21 ± 0.29	3.12 ± 0.26	<0.001
	WBC (10^9^/L)	6.93 (5.54, 9.07)	7.23 (5.56, 9.80)	6.61 (5.48, 8.18)	<0.001
	RBC (10^12^/L)	4.54 ± 0.77	4.45 ± 0.79	4.64 ± 0.72	<0.001
	Neu (10^9^/L)	4.53 (3.49, 6.50)	5.08 (3.71, 7.31)	4.15 (3.30, 5.47)	<0.001
	LYM (10^9^/L)	1.38 (1.01, 1.82)	1.20 (0.84, 1.59)	1.55 (1.21, 2.05)	<0.001
	NLR	3.31 (2.18, 5.72)	4.36 (2.78, 7.49)	2.60 (1.85, 3.91)	<0.001
	CRP (mg/L)	7.50 (3.02, 21.90)	20.87 (12.50, 44.84)	3.00 (1.57, 5.21)	<0.001
	Hb (g/L)	138.07 ± 24.18	135.39 ± 24.97	140.77 ± 22.34	<0.001
	PLT (10^9^/L)	192.00 (148.00, 243.00)	188.00 (141.75, 241.25)	196.00 (156.00, 244.50)	0.057
	Sodium (mmol/L)	141.01 ± 4.43	140.49 ± 4.61	141.74 ± 3.95	<0.001
	Potassium (mmol/L)	3.94 ± 0.60	3.94 ± 0.65	3.94 ± 0.55	0.387
	Chlorine (mmol/L)	102.91 ± 4.68	102.17 ± 4.75	103.73 ± 4.39	<0.001
	ALB (g/dL)	3.67 ± 0.45	3.54 ± 0.44	3.81 ± 0.41	<0.001
	ALT (IU/L)	25.20 (16.70, 42.40)	25.90 (16.00, 45.93)	24.30 (16.80, 40.10)	0.274
	AST (IU/L)	28.60 (20.15, 43.40)	28.95 (20.15, 48.03)	27.70 (20.00, 39.80)	<0.05
	Creatinine (µmol/L)	103.50 (83.20, 134.10)	108.70 (86.28, 140.20)	97.20 (80.10, 123.40)	<0.001
	UA (umol/L)	478.40 (371.75, 588.75)	500.40 (390.55, 610.80)	468.20 (367.45, 567.65)	<0.05
	TC (mmol/L)	3.67 ± 1.00	3.53 ± 0.95	3.81 ± 1.03	<0.001
	GFR (ml/min)	44.05 (32.29, 56.54)	41.77 (29.63, 53.60)	47.28 (35.99, 60.17)	<0.001
	FBG (mmol/L)	5.03 (4.16, 6.50)	5.10 (4.30, 6.98)	4.93 (4.12, 6.11)	<0.05
	TB (umol/L)	15.80 (10.40, 24.60)	16.45 (11.10, 26.23)	15.00 (9.90, 23.15)	<0.05
	HDL (mmol/L)	1.00 ± 0.32	0.94 (0.76, 1.13)	1.01 (0.85, 1.21)	<0.001
	LDL (mmol/L)	2.17 (1.64, 2.76)	2.06 (1.54, 2.69)	2.29 (1.74, 2.83)	<0.001
ECG parameters and cardiac ultrasound index					
	QRS wave (ms)	115.49 ± 29.95	115.86 ± 30.01	115.33 ± 29.78	0.489
	LAd (mm)	42.38 ± 9.39	42.17 ± 9.52	42.77 ± 9.32	0.095
	LVd (mm)	56.30 ± 12.64	55.31 ± 12.42	57.38 ± 12.81	<0.05
	RAd (mm)	51.97 ± 12.55	51.67 ± 12.85	52.40 ± 12.48	0.132
	RVd (mm)	67.36 ± 16.60	67.92 ± 16.17	67.08 ± 16.89	0.240

BMI, body mass index; NYHA, New York Heart Association; WBC, white blood cell; RBC, red blood cell; Neu, neutrophil; LYM, lymphocyte; NLR, neutrophil-to-lymphocyte ratio; Hb, hemoglobin; CRP, C-reactive protein; PLT, platelets; ALB, albumin; ALT, alanine aminotransferase; AST, aspartate aminotransferase; UA, uric acid; TC, total cholesterol; GFR, glomerular filtration rate; FBG, fasting blood-glucose; TB, total bilirubin; HDL, high density lipoprotein; LDL, low density lipoprotein; SBP, systolic blood pressure; DBP, diastolic blood pressure; CRT, cardiac resynchronization therapy; CRT-D, cardiac resynchronization therapy with defibrillator; SGLT2i, sodium-glucose cotransporter 2 inhibitor; ACEI, angiotensin-converting enzyme inhibitor; ARB, angiotensin receptor blocker; ARNI, angiotensin receptor-neprilysin inhibitor; LAd, left atrial diameter; LVd, left ventricular diameter; RAd, right atrial diameter; RVd, right ventricular diameter; lgBNP, log-transformed B-type natriuretic peptide.

The median follow-up time was 768.0 days (IQR: 358.0–1138.0) for the HFpEF subgroup, 737.0 days (IQR: 333.0–1102.0) for the combined HFrEF plus HFmrEF subgroup, and 761.0 days (IQR: 365.5–1102.0) for the HFmrEF subgroup analyzed separately. No significant differences in follow-up duration were observed across subgroups (Kruskal-Wallis test, *p* > 0.05).

### 3.2 Correlation Analysis

**Fig. 2.**
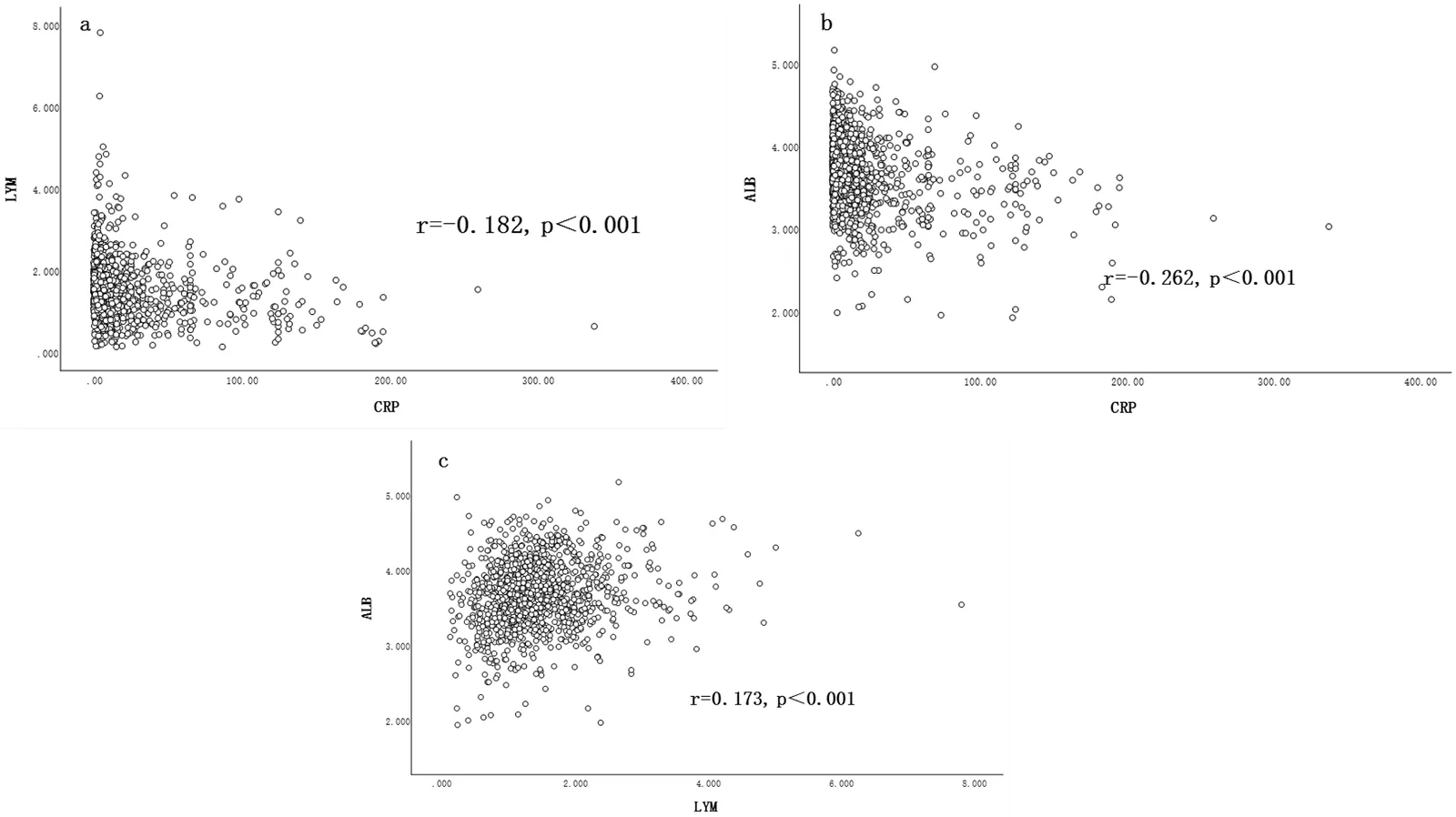
**Correlations between the C-reactive protein (CRP) level, lymphocyte count (LYM), and albumin level (ALB)**. (a) Correlation between LYM and CRP. (b) Correlation between ALB and CRP. (c) Correlation between ALB and LYM.

This study aimed to investigate the prognostic value of the CALLY index for patients with CHF. A correlation analysis of the serum ALB level, C-reactive protein (CRP) level, and the lymphocyte count (LYM) was conducted in patients with chronic heart failure (CHF) using Spearman’s rank correlation test, which is a nonparametric method. The results revealed weak associations among the CRP level, ALB level, and LYM (Fig. 2).

To explore the relationships between CALLY and traditional inflammatory nutritional indicators, we conducted a Spearman correlation analysis. The results (**Supplementary Fig. 2**) revealed that CALLY was moderately negatively correlated with the NLR (r = –0.49), indicating that the higher the inflammatory level was, the lower the CALLY index. CALLY was moderately positively correlated with PNI (r = 0.50), suggesting that the better the nutritional status was, the higher the CALLY index. CALLY was moderately negatively correlated with the CONUT score (r = –0.44), confirming that the more severe the degree of malnutrition was, the lower the CALLY index.

### 3.3 Relationship Between the CALLY Index and Prognosis

The RCS analysis confirmed a significant nonlinear association between the CALLY index and all-cause mortality (*p* for nonlinearity < 0.001). A clinically relevant cutoff value of 0.62 was identified, stratifying the cohort into two groups for primary analysis. For reference, the tertile boundaries of the CALLY index are also marked (T1 and T2, blue dashed lines), corresponding to values of 0.29 and 1.32, respectively. Compared with the reference level, patients with a CALLY index below the cutoff (CALLY <0.62) showed a markedly elevated risk of all-cause mortality, while those with a CALLY index above the cutoff (CALLY ≥0.62) exhibited a reduced mortality risk. The RCS curve demonstrated a steep decline in HR as the CALLY index increased from low values, with the decline gradually attenuating after the cutoff point (Fig. 1).

### 3.4 CALLY and All-Cause Mortality

The Kaplan‑Meier survival analysis revealed that in all heart failure patients and in each subgroup, the all‑cause mortality rate in the low‑CALLY group (CALLY < 0.62) was significantly greater than that in the high‑CALLY group (CALLY ≥0.62) (all *p* values < 0.0001).

During a median follow‑up of 755 days, a total of 562 death events were documented in the total heart failure cohort. In the total heart failure cohort, the low‑CALLY group (n = 598) experienced 383 deaths (event rate: 64.0%), while the high‑CALLY group (n = 595) experienced 179 deaths (event rate: 30.1%). The number of censored cases was 215 in the low‑CALLY group and 416 in the high‑CALLY group. The overall loss to follow‑up rate was 52.9%, with rates of 36.0% in the low‑CALLY group and 69.9% in the high‑CALLY group.

**Fig. 3.**
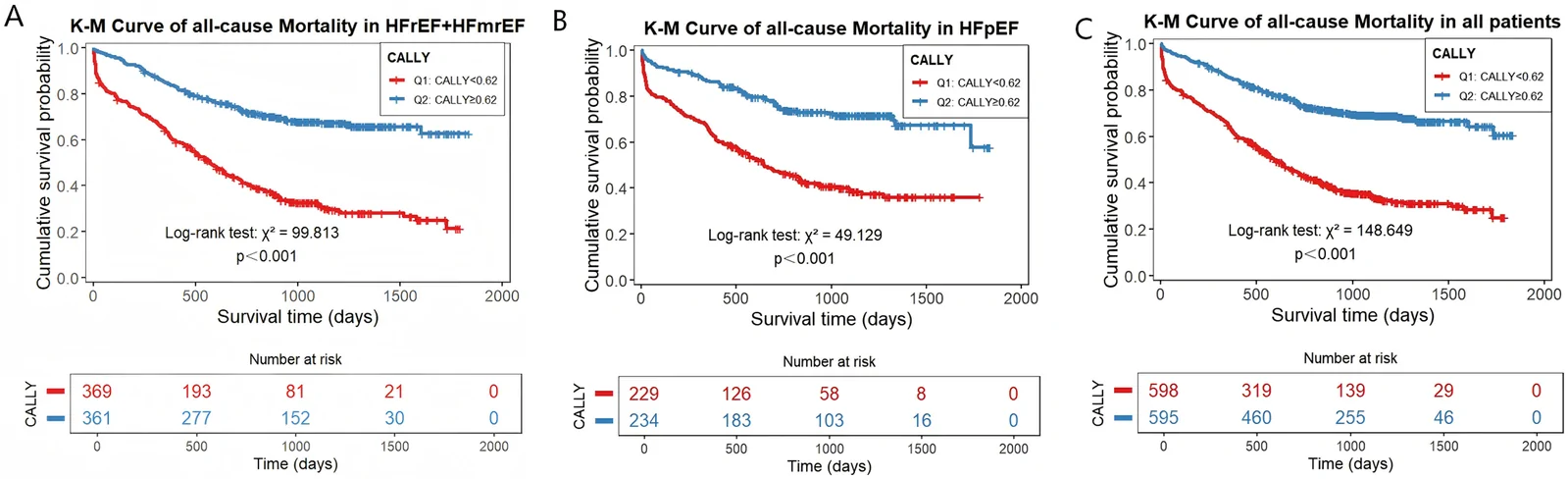
**Kaplan‒Meier survival curves according to the optimal cutoff value of the CALLY index for patients with different subtypes of CHF**. (A) HFrEF + HFmrEF subgroup. (B) HFpEF subgroup. (C) Overall cohort (total population).

In the HFrEF+HFmrEF subgroup (Fig. 3A), the low‑CALLY subgroup (n = 369) and the high‑CALLY subgroup (n = 361) showed significantly different survival curves with respect to follow‑up time, with the low‑CALLY subgroup having a worse prognosis. In this subgroup, 248 deaths occurred in the low‑CALLY group (event rate: 67.2%) and 114 deaths in the high‑CALLY group (event rate: 31.6%).

In the HFpEF subgroup (Fig. 3B), the two groups (n = 229 vs. n = 234) also showed a significant difference in survival (*p* < 0.0001), with 135 deaths in the low‑CALLY group (event rate: 59.0%) and 65 deaths in the high‑CALLY group (event rate: 27.8%). A population‑wide analysis (Fig. 3C; n = 1193) further confirmed that a CALLY index of 0.62 could effectively distinguish high‑risk from low‑risk patients, and that a low CALLY index was closely associated with a high risk of all‑cause mortality. The number of patients at risk at each time interval is displayed below the x‑axis of each Kaplan‑Meier plot (Fig. 3). Detailed survival outcomes by CALLY subgroup are summarized in **Supplementary Table 1**.

### 3.5 The CALLY Index as an Independent Predictor

In a multivariate analysis of the HFrEF plus HFmrEF group, the following variables were statistically significant: classification NYHA (HR: 1.636; 95% CI: 1.294–2.070; *p* < 0.001), pulmonary arterial systolic pressure (HR: 1.008; 95% CI: 1.000–1.016; *p* < 0.05), lgBNP concentration (HR: 2.567; 95% CI: 1.569–4.198; *p* < 0.001), WBC count (HR: 1.034; 95% CI: 1.002–1.067; *p* < 0.05), Hb level (HR: 0.993; 95% CI: 0.988–0.998; *p* < 0.05), GFR (HR: 0.991; 95% CI: 0.981–1.000; *p* < 0.05), UA level (HR: 1.001; 95% CI: 1.000–1.002; *p* < 0.05), and the CALLY index (HR: 2.003; 95% CI: 1.550–2.588; *p* < 0.001). Multivariate analysis revealed that age (HR: 1.037; 95% CI: 1.019–1.056; *p* < 0.001), BMI (HR: 0.936; 95% CI: 0.893–0.980; *p* < 0.05), NYHA class IV (HR: 2.903; 95% CI: 2.111–3.992; *p* < 0.001), lgBNP concentration (HR: 2.575; 95% CI: 1.381–4.799; *p* < 0.05), chlorine concentration (HR: 0.920; 95% CI: 0.884–0.957; *p* < 0.001), AST level (HR: 1.009; 95% CI: 1.004–1.013; *p* < 0.001), FBG(HR: 1.081; 95% CI: 1.041–1.123; *p* < 0.001), and the CALLY index (HR: 1.705; 95% CI: 1.205–2.414; *p* < 0.05) were independent and significant predictors of overall survival (Table 2).

**Table 2.** **Univariate and multivariate analyses of Cox proportional hazards models for patients with HFpEF and HFrEF plus HFmrEF**.

	HFpEF				HFrEF plus HFmrEF			
	Univariable		Multivariable		Univariable		Multivariable	
	HR (95% CI)	*p*	HR (95% CI)	*p*	HR (95% CI)	*p*	HR (95% CI)	*p*
Age	1.045 (1.031, 1.059)	<0.001	1.037 (1.019, 1.056)	<0.001	1.027 (1.018, 1.036)	<0.001	1.007 (0.995, 1.019)	0.245
Sex (ref:Female)	1.129 (0.852, 1.496)	0.397	1.075 (0.774, 1.493)	0.667	0.955 (0.770, 1.185)	0.677	0.972 (0.761, 1.243)	0.824
BMI	0.942 (0.908, 0.978)	<0.05	0.936 (0.893, 0.980)	<0.05	0.944 (0.917, 0.972)	<0.001	0.968 (0.935, 1.002)	0.062
NYHA class(ref:Ⅲ)	2.640 (1.998, 3.487)	<0.001	2.903 (2.111, 3.992)	<0.001	2.292 (1.864, 2.819)	<0.001	1.636 (1.294, 2.070)	<0.001
Pulmonary arterial systolic pressure	1.004 (0.998, 1.009)	0.207			1.013 (1.006, 1.020)	<0.001	1.008 (1.000, 1.016)	<0.05
Smoking (ref:no)	1.138 (0.845, 1.535)	0.395			0.977 (0.789, 1.209)	0.828		
Hypertension	0.867 (0.652, 1.152)	0.325			1.130 (0.920, 1.389)	0.244		
LVEDV	0.996 (0.994, 0.999)	<0.05	0.999 (0.996, 1.001)	0.364	1.001 (1.000, 1.002)	0.181		
LVESV	0.998 (0.995, 1.001)	0.186			1.001 (1.000, 1.003)	0.05		
lgBNP	6.060 (3.645, 10.076)	<0.001	2.575 (1.381, 4.799)	<0.05	6.062 (3.956, 9.289)	<0.001	2.567 (1.569, 4.198)	<0.001
WBC	1.043 (1.010, 1.077)	<0.05	1.003 (0.963, 1.044)	0.901	1.057 (1.032, 1.084)	<0.001	1.034 (1.002, 1.067)	<0.05
LYM	0.760 (0.613, 0.941)	<0.05			0.688 (0.576, 0.822)	<0.001		
Hb	0.989 (0.983, 0.994)	<0.001	0.995 (0.988, 1.001)	0.122	0.991 (0.986, 0.995)	<0.001	0.993 (0.988, 0.998)	<0.05
Sodium	0.943 (0.914, 0.974)	<0.001	1.037 (0.998, 1.077)	0.064	0.935 (0.913, 0.958)	<0.001	0.987 (0.955, 1.020)	0.446
Chlorine	0.925 (0.900, 0.950)	<0.001	0.920 (0.884, 0.957)	<0.001	0.936 (0.914, 0.958)	<0.001	0.976 (0.945, 1.007)	0.131
ALB	0.431 (0.319, 0.580)	<0.001			0.504 (0.392, 0.649)	<0.001		
ALT	1.004 (1.002, 1.007)	<0.001	0.997 (0.992, 1.001)	0.172	1.003 (1.002, 1.004)	<0.001	1.000 (0.998, 1.003)	0.752
AST	1.007 (1.005, 1.009)	<0.001	1.009 (1.004, 1.013)	<0.001	1.004 (1.002, 1.005)	<0.001	1.001 (0.999, 1.004)	0.345
Urea	1.049 (1.027, 1.072)	<0.001	1.008 (0.973, 1.045)	0.657	1.057 (1.040, 1.074)	<0.001	0.985 (0.961, 1.010)	0.237
GFR	0.976 (0.968, 0.984)	<0.001	0.994 (0.982, 1.006)	0.333	0.9747 (0.967, 0.980)	<0.001	0.991 (0.981, 1.000)	<0.05
FBG	1.073 (1.040, 1.108)	<0.001	1.081 (1.041, 1.123)	<0.001	1.040 (1.011, 1.070)	<0.05	1.015(0.981, 1.051)	0.394
UA	1.001 (1.000, 1.002)	<0.05	1.000 (0.999, 1.001)	0.689	1.002 (1.001, 1.002)	<0.001	1.001 (1.000, 1.002)	<0.05
TC	0.801 (0.684, 0.939)	<0.05	1.039 (0.873, 1.236)	0.668	0.796 (0.710, 0.893)	<0.001	0.957 (0.847, 1.082)	0.484
CRP	1.011 (1.009, 1.013)	<0.001			1.010 (1.008, 1.013)	<0.001		
Fib	1.124 (1.011, 1.250)	<0.05	1.003 (0.893, 1.126)	0.96	1.022 (0.941, 1.109)	0.609		
CALLY (ref:high CALLY)	2.768 (2.057, 3.726)	<0.001	1.705 (1.205, 2.414)	<0.05	2.952 (2.362, 3.688)	<0.001	2.003 (1.550, 2.588)	<0.001

LVEDV, left ventricular end diastolic volume; LVESV, left ventricular end-systolic volume.

In the HFmrEF subgroup, in the multivariate analysis, the CALLY index (HR: 2.679; 95% CI: 1.707–4.205; *p* < 0.001) were independent and significant predictors of overall survival (Table 3).

**Table 3.** **Univariate and multivariate analyses of Cox proportional hazards models for patients with HFmrEF**.

	Univariable		Multivariable	
	HR (95% CI)	*p*	HR (95% CI)	*p*
Age	1.035 (1.017, 1.052)	<0.001	1.015 (0.993, 1.037)	0.190
Sex (ref:Female)	0.822 (0.567, 1.192)	0.301	0.963 (0.649, 1.428)	0.851
BMI	0.967 (0.918, 1.018)	0.200	0.974 (0.918, 1.033)	0.376
NYHA class (ref:Ⅲ)	2.206 (1.519, 3.203)	<0.001	1.530 (1.191, 2.362)	<0.05
Pulmonary arterial systolic pressure	1.009 (0.996, 1.021)	0.177		
Smoking (ref:no)	0.881 (0.587, 1.323)	0.542		
Hypertension	1.055 (0.722, 1.542)	0.781		
LVEDV	0.998 (0.995, 1.000)	0.108		
LVESV	0.999 (0.995, 1.003)	0.579		
lgBNP	3.587 (1.611, 7.986)	<0.05	1.158 (0.488, 2.748)	0.740
WBC	1.088 (1.048, 1.129)	<0.001	1.050 (1.004, 1.098)	<0.05
LYM	0.697 (0.490, 0.990)	<0.05		
Hb	0.995 (0.986, 1.003)	0.214		
Sodium	0.910 (0.869, 0.952)	<0.001	0.951 (0.899, 1.006)	0.080
Chlorine	0.919 (0.877, 0.964)	<0.001	0.962 (0.910, 1.017)	0.168
ALB	0.585 (0.380, 0.902)	<0.05		
ALT	1.002 (1.001, 1.004)	<0.05	1.001 (0.997, 1.004)	0.731
AST	1.004 (1.002, 1.005)	<0.001	1.002 (0.998, 1.005)	0.364
Urea	1.047 (1.012, 1.083)	<0.05	0.998 (0.952, 1.046)	0.935
GFR	0.979 (0.968, 0.991)	<0.001	0.992 (0.976, 1.008)	0.322
FBG	1.061 (1.010, 1.116)	<0.05	0.983 (0.926, 1.043)	0.560
UA	1.001 (0.999, 1.002)	0.343	1.000 (0.999, 1.001)	0.537
TC	0.866 (0.717, 1.046)	0.136		
CRP	1.009 (1.005, 1.013)	<0.001		
Fib	0.984 (0.865, 1.119)	0.808		
CALLY (ref:high CALLY)	3.155 (2.073, 4.803)	<0.001	2.679 (1.707, 4.205)	<0.001

In the Cox multivariate analysis of all patients with CHF, the following variables were found to be independent predictors of prognosis in patients with CHF: age (HR: 1.015; 95% CI: 1.006–1.025; *p* < 0.05), BMI (HR: 0.961; 95% CI: 0.936–0.988; *p* < 0.05), NYHA classification (HR: 1.870; 95% CI: 1.554–2.249; *p* < 0.001), lgBNP concentration (HR: 2.522; 95% CI: 1.768–3.598; *p* < 0.001), WBC count (HR: 1.025; 95% CI: 1.000–1.051; *p* < 0.05), Hb (HR: 0.993; 95% CI: 0.989–0.997; *p* < 0.05), chlorine concentration (HR: 0.957; 95% CI: 0.934–0.980; *p* < 0.001), AST level (HR: 1.002; 95% CI: 1.000–1.005;* p* < 0.05), and GFR(HR: 0.992; 95% CI: 0.985–0.999; *p* < 0.05), FBG (HR: 1.030; 95% CI: 1.004–1.057; *p* < 0.05), UA (HR: 1.001; 95% CI: 1.000–1.001; *p* < 0.05), the CALLY index (HR: 1.913; 95% CI: 1.560–2.345; *p* < 0.001) (Table 4).

**Table 4.** **Univariate and multivariate analyses of Cox proportional hazards models for all the patients**.

	Univariable		Multivariable	
	HR (95% CI)	*p*	HR (95% CI)	*p*
Age	1.031 (1.023, 1.038)	<0.001	1.015 (1.006, 1.025)	<0.05
Sex (ref:Female)	1.035 (0.872, 1.228)	0.694	0.980 (0.808, 1.189)	0.838
BMI	0.944 (0.922, 0.965)	<0.001	0.961 (0.936, 0.988)	<0.05
NYHA class (ref:Ⅲ)	2.427 (2.056, 2.865)	<0.001	1.870 (1.554, 2.249)	<0.001
Pulmonary arterial systolic pressure	1.006 (1.002, 1.010)	<0.05	1.002 (0.998, 1.006)	0.364
Smoking (ref:no)	1.043 (0.877, 1.240)	0.635		
Hypertension	1.009 (0.855, 1.192)	0.914		
LVEDV	1.000 (0.999, 1.001)	0.891		
LVESV	1.001 (1.000, 1.002)	0.091		
lgBNP	5.264 (3.867, 7.164)	<0.001	2.522 (1.768, 3.598)	<0.001
WBC	1.051 (1.031, 1.072)	<0.001	1.025 (1.000, 1.051)	<0.05
LYM	0.717 (0.625, 0.823)	<0.001		
Hb	0.990 (0.987, 0.994)	<0.001	0.993 (0.989, 0.997)	<0.001
Sodium	0.938 (0.920, 0.956)	<0.001	1.005 (0.980, 1.030)	0.698
Chlorine	0.930 (0.914, 0.947)	<0.001	0.957 (0.934, 0.980)	<0.001
ALB	0.484 (0.400, 0.586)	<0.001		
ALT	1.003 (1.002, 1.004)	<0.001	1.000 (0.998, 1.002)	0.933
AST	1.004 (1.003, 1.005)	<0.001	1.002 (1.000, 1.005)	<0.05
Urea	1.054 (1.041, 1.068)	<0.001	0.992 (0.973, 1.011)	0.405
GFR	0.975 (0.970, 0.980)	<0.001	0.992 (0.985, 0.999)	<0.05
FBG	1.054 (1.032, 1.077)	<0.001	1.030 (1.004, 1.057)	<0.05
UA	1.001 (1.001, 1.002)	<0.001	1.001 (1.000, 1.001)	<0.05
TC	0.802 (0.731, 0.880)	<0.001	0.952 (0.862, 1.051)	0.33
CRP	1.011 (1.009, 1.012)	<0.001		
Fib	1.051 (0.986, 1.121)	0.127		
CALLY (ref:high CALLY)	2.881 (2.411, 3.442)	<0.001	1.913 (1.560, 2.345)	<0.001

To assess the robustness of the dichotomized findings and to address concerns about information loss, we repeated the analysis after categorizing CALLY into tertiles (lowest, middle, and highest) in the overall population as well as in the HFpEF and HFrEF plus HFmrEF subgroups. In the overall population, compared with the highest tertile, the middle tertile showed a progressively increased risk of mortality (HR = 1.938, 95% CI: 1.257–2.988), and the lowest tertile showed the highest risk (HR = 1.984, 95% CI: 1.622–2.428), with a significant trend (*p* for trend < 0.05). This graded association is consistent with the direction observed in the dichotomized analysis (CALLY <0.62 vs. ≥0.62). Similar patterns were observed in the HFpEF subgroup (middle tertile: HR = 2.025, 95% CI: 1.186–3.456; lowest tertile: HR = 2.167, 95% CI: 1.679–2.798; *p* for trend < 0.05) and in the HFrEF plus HFmrEF subgroup (middle tertile: HR = 2.083, 95% CI: 1.200–3.615; lowest tertile: HR = 2.112, 95% CI: 1.656–2.693; *p* for trend < 0.05). These findings confirm that the association between CALLY and mortality is not an artifact of the specific dichotomous cutoff and remains robust across different stratification methods and clinical subgroups (Table 5).

**Table 5.** **Sensitivity analysis: association between CALLY tertiles and clinical outcome**.

Population	Category	Patients (N)	HR (95% CI)	*p*-value
Total	T3 (High): reference	755		
	T1 (Low)	397	1.984 (1.622, 2.428)	<0.001
	T2 (Medium)	41	1.938 (1.257, 2.988)	<0.05
HFpEF	T3 (High): reference	295		
	T1 (Low)	152	2.167 (1.679, 2.798)	<0.001
	T2 (Medium)	16	2.025 (1.186, 3.456)	<0.05
HFrEF plus HFmrEF	T3 (High): reference	460		
	T1 (Low)	245	2.112 (1.656, 2.693)	<0.001
	T2 (Medium)	25	2.083 (1.200, 3.615)	<0.05

### 3.6 Predictive Ability of the CALLY Index

To more thoroughly investigate the predictive ability of the CALLY index, we constructed four basic models.

Model 1 was adjusted for age and sex. Model 2 was derived by adding the NYHA classification, BMI, and GFR to Model 1. Model 3 was then developed by further incorporating lgBNP, chloride, and sodium concentrations into Model 2. Finally, the CALLY index was included in Model 3 to construct the updated Model 4.

**Fig. 4.**
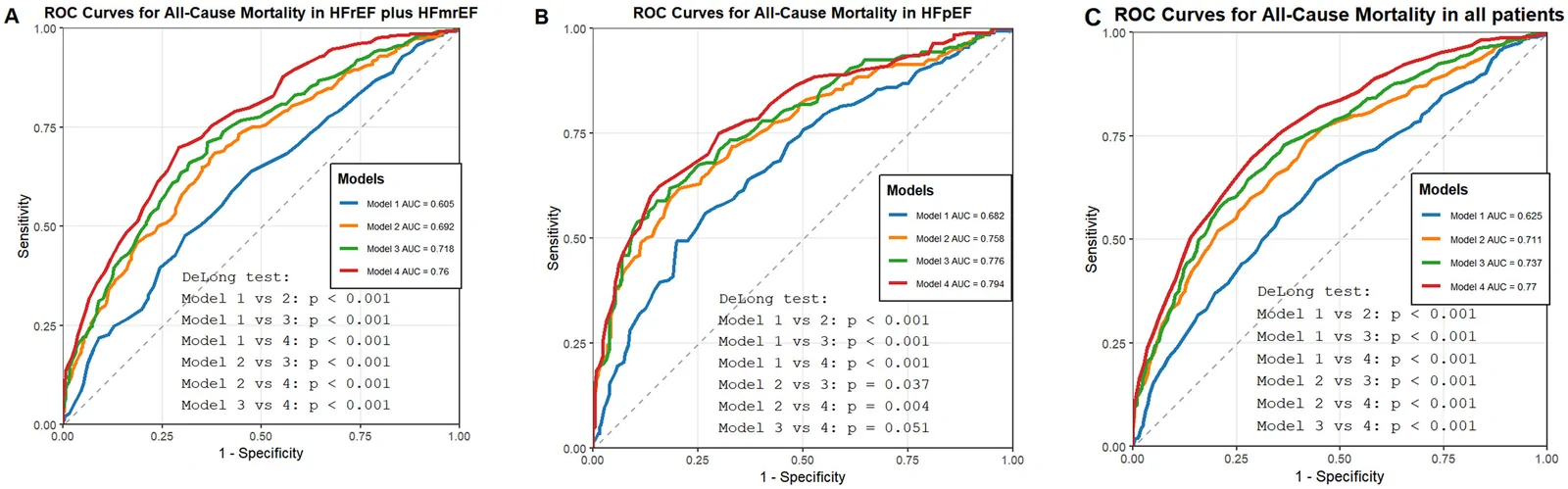
**ROC curves of different models used to predict all-cause mortality in patients with different subtypes of CHF**. Ref: low CALLY. Model 1: Adjusted for Age and Sex. Model 2: Adjusted for the variables included in Model 1 and NYHA class, BMI, and GFR. Model 3: Adjusted for the variables included in Model 2 and lgBNP, sodium, and chlorine concentrations. Model 4: Adjusted for the variables included in Model 3 and the CALLY index. (A) HFrEF + HFmrEF subgroup. (B) HFpEF subgroup. (C) Total population.

Receiver operating characteristic (ROC) curves were constructed to evaluate the predictive performance of the four models for all-cause mortality in different subgroups of heart failure patients and in the overall cohort for all-cause mortality (Fig. 4A–C).

In the HFrEF plus HFmrEF subgroup (Fig. 4A), the area under the ROC curve (AUC) values were 0.605 (Model 1), 0.692 (Model 2), 0.718 (Model 3), and 0.760 (Model 4). The DeLong test revealed highly significant differences between all pairwise model comparisons (all *p* < 0.001), indicating that Model 4 had better discrimination ability than the other three models did.

In the HFpEF subgroup (Fig. 4B), the AUC values were 0.682 (Model 1), 0.758 (Model 2), 0.776 (Model 3), and 0.794 (Model 4). Model 4 achieved the highest AUC value, and significant differences were observed in all comparisons involving Model 1 (all *p* < 0.001). Model 3 was also superior to Model 2 (*p* = 0.037), and Model 4 was also superior to Model 2 (*p* = 0.004), while there was no statistically significant difference between Model 3 and Model 4 (*p* = 0.051).

In all patients (Fig. 4C), the AUC values were 0.625 (Model 1), 0.711 (Model 2), 0.737 (Model 3), and 0.770 (Model 4). The AUC value of Model 4 was again the highest, and the DeLong test revealed highly significant differences between all pairwise model comparisons (all *p* < 0.001).

Overall, these results indicate that compared with most other models, Model 4 consistently has the strongest predictive ability to predict all-cause mortality in all the subgroups and the entire patient population and has significant statistical advantages.

In addition, we calculated the sensitivity and specificity of the four models. As shown in Table 6, for patients with HFpEF, Model 4 had the highest sensitivity, with a sensitivity of 0.620, while Model 4 had a specificity of 0.852, with the greatest heterogeneity among the four models. For patients with HFrEF plus HFmrEF patients, Model 4 had the highest specificity, with a specificity of 0.712. For all patients with CHF, Model 4 had a sensitivity of 0.724, the best of the four models.

**Table 6.** **The predictive value of the CALLY index for the HFpEF group, HFrEF plus HFmrEF group, and all patients according to the four models**.

	HFpEF					HFrEF plus HFmrEF					All patients with CHF				
	AUC	Sensitivity	Specificity	95% CI	*p* value	AUC	Sensitivity	Specificity	95% CI	*p* value	AUC	Sensitivity	Specificity	95% CI	*p* value
Model 1	0.682	0.560	0.734	(0.632, 0.731)	<0.001	0.605	0.610	0.565	(0.564, 0.646)	<0.001	0.625	0.642	0.558	(0.593, 0.656)	<0.001
Model 2	0.758	0.600	0.817	(0.713, 0.803)	<0.001	0.692	0.687	0.625	(0.656, 0.732)	<0.001	0.711	0.595	0.731	(0.683, 0.741)	<0.001
Model 3	0.776	0.620	0.821	(0.733, 0.820)	<0.001	0.718	0.715	0.636	(0.683, 0.757)	<0.001	0.737	0.588	0.784	(0.710, 0.766)	<0.001
Model 4	0.794	0.620	0.852	(0.752, 0.836)	<0.001	0.760	0.701	0.712	(0.728, 0.796)	<0.001	0.770	0.724	0.694	(0.744, 0.797)	<0.001

Model 1: Adjusted for age and sex. Model 2: Adjusted for the variables included in Model 1 and NYHA class, BMI and GFR. Model 3: Adjusted for the variables included in Model 2 and lgBNP, sodium, chlorine concentrations. Model 4: Adjusted for the variables included in Model 3 and the CALLY index.

**Fig. 5.**
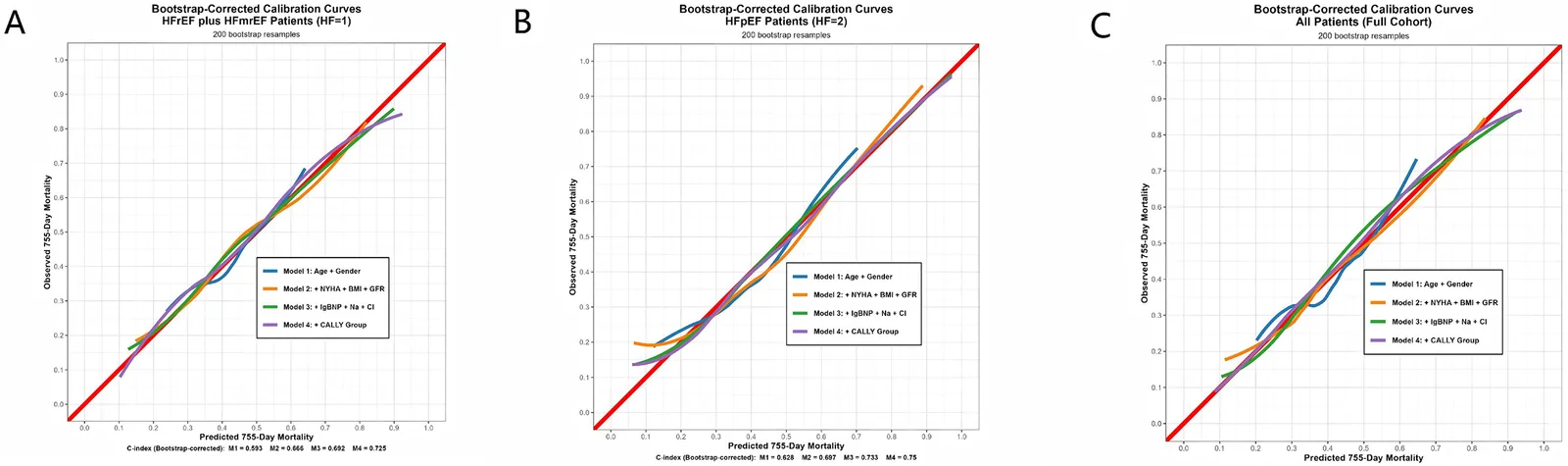
**Bootstrap-corrected calibration curves**. (A) HFrEF + HFmrEF subgroup. (B) HFpEF subgroup. (C) Total population.

The calibration curve reveals a good consistency between the predicted probability and the observed results. In the entire population of heart failure patients, the C-index generated by bootstrap internal validation (200 samples) increased from 0.600 (Model 1: age + sex) to 0.737 (Model 4: + CALLY group). Similar incremental improvements were also observed in subgroups of HFpEF (0.628 to 0.750) and HFrEF plus HFmrEF (0.593 to 0.725) (Fig. 5A–C). The calibration curve shows that Model 4 has the best fit to the ideal diagonal in all risk stratifications and subgroups.

**Fig. 6.**
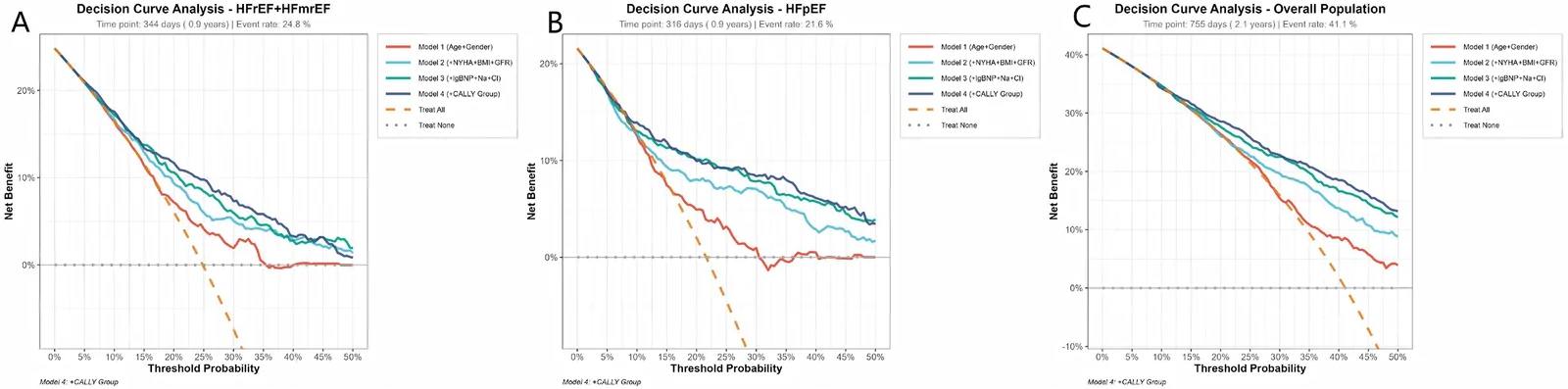
**Decision curve analysis**. (A) HFrEF + HFmrEF subgroup. (B) HFpEF subgroup. (C) Total population.

Decision curve analysis (DCA) was performed to evaluate the clinical net benefit of each model across different subgroups and the overall population. As illustrated in Fig. 6, compared with the extreme strategies of “Treat All” or “Treat None” strategies, all the predictive models yielded superior net benefits across a wide range of threshold probabilities. Notably, Model 4 consistently exhibited the greatest net benefit in the HFrEF plus HFmrEF subgroup (Fig. 6A), HFpEF subgroup (Fig. 6B), and overall population (Fig. 6C), outperforming the other three models throughout most clinically relevant threshold ranges. In the HFrEF plus HFmrEF subgroup (follow-up: 344 days; event rate: 24.8%), Model 4 showed the most pronounced advantage within the threshold probability range of 15%–45%. In the HFpEF subgroup (follow-up: 316 days; event rate: 21.6%), Model 4 maintained a superior net benefit between 10% and 40%. For the overall population (follow-up: 755 days; event rate: 41.1%), Model 4 demonstrated the greatest net benefit across the entire threshold spectrum, confirming its robust predictive performance and clinical utility in long-term risk stratification.

These findings indicate that compared with traditional biomarker-based models, the model that incorporated the CALLY index has greater prognostic accuracy and clinical decision-making value. The consistent superiority of Model 4 across different ejection fraction subtypes further supports its generalizability to the broader heart failure population, making it a promising tool for individualized risk assessment and targeted intervention strategies.

### 3.7 Model Diagnostics

The CALLY index satisfied the proportional hazards assumption (*p* = 0.822), whereas the lgBNP concentration (*p* = 0.0003), sodium concentration (*p* = 0.0077), and the GFR (*p* = 0.0070) violated the PH assumption (**Supplementary Table 2**).

## 4. Discussion

This study demonstrates that the CALLY index is an independent predictor of all-cause mortality in patients with heart failure, with differential prognostic value across HFrEF, HFmrEF, and HFpEF phenotypes. Multivariate Cox regression analysis revealed that the CALLY index was independently associated with the risk of all-cause mortality, and this association was consistent in the combined HFrEF+HFmrEF group and the HFpEF group. The model incorporating the CALLY index demonstrated superior predictive performance and clinical utility, as further supported by the results of the calibration and decision curve analyses.

CHF is a multifactorial systemic disease in which cardiac injury leads to the activation of a network of neurohumoral, cellular, and molecular mechanisms to maintain physiological function. This pathophysiological cascade results in hemodynamic overload, sustained sympathetic hyperactivity, and circulatory redistribution, ultimately manifesting as diverse clinical manifestations mediated by immune system dysregulation [14]. Myocardial injury initiates the innate immune response by activating proinflammatory cytokines (such as TNF-α and IL-6), chemokines, and complement components, leading to endothelial cell activation and the consequent infiltration of monocytes and neutrophils. Crucially, the adaptive immune system becomes engaged via myocardial recruitment of T lymphocytes and B lymphocytes, driving cardiac remodeling processes that culminate in adverse structural changes [4]. In addition, Walter J. Paulus and colleagues [15] proposed a novel mechanistic framework for HFpEF pathophysiology, positing that HFpEF-associated comorbidities (e.g., hypertension and diabetes) induce coronary microvascular endothelial inflammation. This inflammatory cascade drives myocardial dysfunction and adverse remodeling through chronic inflammatory signaling pathways, thereby establishing systemic inflammation as a pivotal pathogenic determinant of HFpEF development [15]. Emerging evidence highlights that myocardial energy metabolism relies fundamentally on adequate nutritional substrate availability to sustain cardiac mechanical efficiency. Heart failure progression induces a catabolic state characterized by (1) reduced nutrient intake due to anorexia, (2) intestinal barrier dysfunction secondary to congestion-induced edema, (3) increased resting energy expenditure, and (4) systemic inflammation-induced hypercatabolism mediated by proinflammatory cytokines, such as TNF-α and IL-6. This malnutrition‒inflammation axis creates a self-perpetuating cycle that exacerbates cardiac dysfunction and limits compensatory mechanisms [16]. The CALLY index integrates three readily available laboratory parameters: albumin, a marker of nutritional reserve and a negative acute-phase reactant; lymphocyte count, reflecting adaptive immune competence; and C-reactive protein (CRP), a classic indicator of systemic inflammation. By combining these three components, CALLY captures the dual burden of nutritional depletion and immune-inflammatory activation in a single composite metric, which may provide a more comprehensive risk assessment than any single parameter alone.

The 2021 European Society of Cardiology Heart Failure Guidelines stratify heart failure patients into distinct phenotypes on the basis of LVEF: HFrEF (EF ≤40%), HFpEF (EF ≥50%), and HFmrEF (40% < EF < 49%) [3]. Through network analysis incorporating 92 biomarkers across diverse pathophysiological domains, Jasper Tromp and colleagues [17] reported that biological pathways in HFrEF are linked to DNA-binding transcription factor activity, cellular protein metabolic processes, and the regulation of nitric oxide production, in contrast, HFpEF patients exhibit distinct pathways related to cytokine signaling, extracellular matrix remodeling, and inflammatory processes [17]. In patients with HFmrEF, biomarker levels were intermediate between those in patients with HFrEF and those in patients with HFpEF, with biomarker interactions between cardiac tractions and inflammatory markers [18]. Second, HFpEF and HFrEF differ significantly in terms of intracellular calcium regulation and excitation‒contraction (EC) coupling, as well as in β-adrenergic sensitivity [19]. Therefore, it is necessary to analyze heart failure patients with different ejection fractions.

Notably, the prognostic value of the CALLY index has been primarily studied in Asian populations, with limited Western data. A US-based study reported an inverse association between the CALLY index and the incidence of angina, which is consistent with findings from Chinese STEMI cohorts and supports the role of inflammatory-nutritional markers in cardiovascular prognosis [12,20]. Heart failure phenotypes may differ between Asian and Western populations: Asian patients present at a younger age [21], with a higher comorbidity burden in patients with HFrEF but comparable profiles in patients with HFpEF [22,23]. These differences may influence the prognostic performance of the CALLY index across ethnicities, warranting validation in multinational cohorts.

Albumin synthesis begins in hepatocytes as preproalbumin, which is subsequently modified posttranslationally within the lumen of the endoplasmic reticulum and ultimately matures in the Golgi apparatus [24]. Albumin plays critical physiological roles through antioxidant activity, anti-inflammatory modulation, anticoagulant effects, and colloid osmotic pressure regulation while also facilitating the transport of endogenous metabolites and exogenous xenobiotics. Although albumin is classified as a negative-phase reactant, its synthesis is suppressed by proinflammatory cytokines (e.g., IL-6 and TNF-α), and it paradoxically exhibits anti-inflammatory properties via the selective inhibition of cytokine-induced endothelial activation pathways [25,26]. In individuals with acute decompensated heart failure (ADHF), low albumin levels are linked to gastrointestinal protein loss resulting from visceral stasis; elevated interstitial fluid within intestinal villi disrupts capillary integrity, promoting macromolecular (including albumin) and lymphocyte translocation into the gastrointestinal lumen. This pathophysiological cascade results in concurrent hypoalbuminemia and lymphocytopenia [27]. J.G. Carr et al. [28] demonstrated that severe congestive heart failure patients with hypoalbuminemia exhibited elevated right atrial pressure and severe tricuspid regurgitation. Albumin serves as a composite biomarker reflecting malnutrition, comorbidity burden, and systemic inflammation [29]. In a 2012 respective cohort study, Liu et al. [30] stratified participants into hypoalbuminemia (albumin concentration <3.4 g/dL) and normoalbuminemia groups on the basis of albumin levels at hospital admission. At the 1-year follow-up, hypoalbuminemia was independently associated with increased all-cause mortality in HFpEF patients, establishing that the concentration of 1-year follow-up, hypoalbuminemia was independently associated with increased all-cause mortality in HFpEF patients, establishing that the concentration of albumin is a robust prognostic predictor in HFpEF patients [30]. In the COAPT (Cardiovascular Outcomes Assessment of Patients with Functional Mitral Regurgitation by MitraClip Percutaneous Therapy) trial, Kent Y. Feng and his colleagues [31] compared the impact of transcatheter edge-to-edge repair (TEER) using the MitraClip system combined with guideline-directed medical therapy (GDMT) versus GDMT alone on survival outcomes in patients with symptomatic heart failure and moderate-to-severe secondary mitral regurgitation (SMR). a low baseline serum albumin concentration was independently correlated with decreased four-year survival among patients with heart failure and severe secondary mitral regurgitation enrolled in the COAPT trial. Additionally, treatment with TEER was associated with lower rates of heart failure-related hospitalizations [31].

C-reactive protein (CRP) is a classic acute-phase protein primarily produced by hepatocytes, endothelial cells, and adipose tissue in response to stimulation by interleukin-6 (IL-6) and IL-1β, and it plays a critical role in the development and progression of cardiovascular diseases [32]. Following myocardial injury, CRP binds to pattern recognition receptors on innate immune cells, triggering humoral immune responses characterized by proinflammatory cytokine release (e.g., TNF-α and IL-8), chemokine upregulation, and complement system activation [33]. Elevated CRP levels are driven by comorbid conditions including obesity, diabetes mellitus, hypertension, and chronic kidney disease, which induce systemic inflammation through multiple pathways. This chronic inflammatory environment triggers abnormal laminin‒myosin interactions, upregulates inducible nitric oxide synthase expression in cardiomyocytes, and impairs the ability of the heart to manage unfolded proteins, culminating in myocardial fibrosis, cardiac hypertrophy, increased left ventricular diastolic stiffness, and progression to clinical heart failure [34]. Importantly, CRP directly inhibits endothelial nitric oxide synthase (eNOS) activity and exacerbates endothelial dysfunction *in vitro* [35]. The 2002 study by J.L. Alonso-Martínez et al. [36] first demonstrated the independent association of the CRP level with increased readmission and mortality rates in heart failure patients. Building on this foundation, the 2024 BASELV prospective multicenter diagnostic study evaluated model performance by incorporating the CRP level into the MEESSI-AHF risk score. The expanded model significantly improved the 30-day mortality prediction accuracy [37]. In the 2019 CANTOS trial, Brendan M. Everett et al. [38] randomized 10,061 postmyocardial infarction patients with baseline hs-CRP levels ≥2 mg/L to receive canakinumab (interleukin-1β inhibitor) or placebo. The findings demonstrated that patients hospitalized for heart failure generally presented with elevated baseline concentrations of high-sensitivity C-reactive protein (hs-CRP) and that canakinumab treatment was associated with a reduced risk of the composite endpoint of heart failure hospitalization or heart failure-related mortality. These results suggest a potential therapeutic role for anti-inflammatory interventions in mitigating the risk of heart failure progression [38].

Reduced relative lymphocyte levels are an independent predictor of unfavorable outcomes in patients with congestive heart failure [39]. Mechanistic studies have revealed that lymphopenia in HF arises from synergistic interactions between chronic inflammation, malnutrition, intestinal lymphatic congestion, and β-adrenergic hyperactivity. Following sustained myocardial injury, the innate immune system is activated, which in turn triggers an adaptive immune response by recruiting B and T cells to the damaged myocardium. This process promotes the release of cellular components involved in the immune response through the recruitment of T cells (e.g., TH cells and Treg cells), B cells, mast cells, natural killer cells (NK cells), and dendritic cells [33]. Notably, the expression of myocardial GRK2, an enzyme that regulates β-adrenergic receptors (β-AR) in the heart, was upregulated in patients with heart failure, and the levels of circulating lymphocyte GRK2 correlated with myocardial GRK2 activity, suggesting the presence of systemic immune‒cardiac interactions [40]. Lymphocytopenia also results from intestinal barrier dysfunction, facilitating bacterial translocation and endotoxin-mediated immune activation [41,42]. Additionally, relative lymphocytopenia is inversely associated with jugular venous pressure, suggesting that systemic venous hypertension and visceral congestion may underlie the occurrence of lymphocytopenia in heart failure patients. Lymphocytopenia in patients with heart failure may result from autophagy and apoptosis of activated lymphocytes, while heightened sympathetic activity and elevated oxidative stress or proinflammatory conditions contribute to lymphocyte depletion by triggering programmed cell death [43].

Recently, an increasing number of studies have focused on the correlation between inflammation/nutrition-related markers and the prognosis of cardiovascular disease. In previous studies, the CALLY index has been used to assess the prognosis of patients with gastric, colorectal, hepatocellular, and esophageal squamous cell carcinoma; in addition, the CALLY index independently predicts short- and long-term MACEs in patients with STEMI after PCI [8,12,13,44,45]. Our study illustrates for the first time the relationship between CALLY levels and all-cause mortality in CHF patients with different ejection fractions, and given that these biomarkers are easily accessible and can be measured without the need for specialized instruments, it is reasonable to use the CALLY index as a tool for the initial assessment of prognosis in CHF patients with different ejection fractions in future studies.

## 5. Limitations

Several limitations should be acknowledged. First, this was a retrospective, single-center study of hospitalized patients with advanced heart failure (NYHA class III or IV), which limits generalizability to outpatients with milder disease or to other healthcare settings. Second, telephone follow-up may have introduced information bias, particularly for outcome ascertainment. Third, only baseline CALLY components were analyzed; longitudinal measurements were not available, precluding assessment of dynamic changes in inflammatory and nutritional status over time. Additionally, laboratory values were obtained at hospital admission, and although patients with active infection were excluded, the potential influence of acute decompensation status on these measurements cannot be fully excluded. Fourth, the CALLY index was dichotomized based on a cutoff derived from restricted cubic spline analysis within the same cohort without external validation. Although internal validation via bootstrap resampling demonstrated adequate model calibration and sensitivity analyses using tertiles yielded consistent results, the potential for overfitting and information loss remains. Fifth, the study population was exclusively Chinese, which limits generalizability to other ethnic populations. Finally, comparisons with established heart failure risk scores (e.g., MAGGIC, Seattle Heart Failure Model) were not performed, leaving the incremental prognostic value of CALLY relative to existing tools to be explored in future studies. Sixth, despite careful adjustment for major confounders and exclusion of patients with active infection or malignancy, the potential for residual confounding remains. Unmeasured factors such as frailty, detailed nutritional status, inflammatory burden, and medication adherence may have influenced the observed associations.

## 6. Future Directions

First, given that the optimal CALLY cutoff (0.62) was derived from a single-center cohort without external validation, the generalizability of our findings to other populations remains uncertain. Future multicenter prospective studies should include diverse populations and outpatients with mild heart failure to externally validate the CALLY index across different clinical settings. Second, serial CALLY measurements should be incorporated to assess its dynamic prognostic value. Third, the CALLY index should be compared against validated risk models to determine its added predictive utility. Finally, alternative follow-up methods (e.g., in-person visits or electronic health record linkage) should be considered to minimize information bias.

## 7. Conclusions

Among CHF patients in this cohort, a lower CALLY index was independently associated with higher all-cause mortality across different ejection fraction categories. These findings suggest that CALLY may serve as a potential risk marker, pending external validation in diverse populations.

## Data Availability

The data analyzed in this study contain sensitive clinical information, including patient records and geographic locations. Even after anonymization, there remains a potential risk of re-identification. Therefore, the original data cannot be made publicly accessible at this time. To ensure the reproducibility of our findings, we have provided a detailed description of the data processing procedures in the manuscript. Should readers or reviewers require further verification, we are happy to cooperate and provide additional support. Requests for access to the datasets may be directed to the corresponding author via the following email: ydyyclx@163.com.
