# Supplementary material for: Clinical Prognostic Impact of CALLY in Heart Failure Patients With Different Ejection Fractions: A Retrospective Study
Source: Rev Cardiovasc Med. 2026 Aug 25;27(8):49108. doi: 10.31083/RCM49108 (PMC13540055; doi:10.31083/RCM49108)
Supplement: Supplementary file 1 [file 2153-8174-27-8-49108-s1.zip › Supplementary Material.docx]

Supplementary Figure 1. Patient eligibility flowchart

Supplementary Figure 2 The correlation between the CALLY level, PNI level, CONUT score and NLR level.

Supplementary Table 1 Survival outcomes stratified by CALLY groups

| Population | CALLY Group | n | Death events, n (%) | Censored, n (%) | Loss to follow-up, n (%) |
| --- | --- | --- | --- | --- | --- |
| Total heart failure cohort | Low (<0.62) | 598 | 383 (64.0) | 215 (36.0) | 215 (36.0) |
|  | High (≥0.62) | 595 | 179 (30.1) | 416 (69.9) | 416 (69.9) |
|  | Overall | 1,193 | 562 (47.1) | 631 (52.9) | 631 (52.9) |
| HFrEF plus HFmrEF |  |  |  |  |  |
|  | Low (<0.62) | 369 | 248 (67.2) | 121 (32.8) | 121 (32.8) |
|  | High (≥0.62) | 361 | 114 (31.6) | 247 (68.4) | 247 (68.4) |
|  | Overall | 730 | 362 (49.6) | 368 (50.4) | 368 (50.4) |
| HFpEF |  |  |  |  |  |
|  | Low (<0.62) | 229 | 135 (59.0) | 94 (41.0) | 94 (41.0) |
|  | High (≥0.62) | 234 | 65 (27.8) | 169 (72.2) | 169 (72.2) |
|  | Overall | 463 | 200 (43.2) | 263 (56.8) | 263 (56.8) |
| CALLY groups were defined based on the optimal cutoff (0.62) derived from restricted cubic spline analysis: low CALLY (<0.62) and high CALLY (≥0.62). Loss to follow‑up was defined as patients who were alive at the end of the study period without experiencing the outcome of interest or who were lost before study completion. Event rates and censored rates are presented as n (% of the group total). | | | | | |

Supplementary Table 2 Proportional Hazards Assumption Test Results.

| Variable | Chisq | df | p_value | PH_Status |
| --- | --- | --- | --- | --- |
| Age | 0.147 | 1 | 0.7015 | Satisfied |
| Gender | 0.089 | 1 | 0.7656 | Satisfied |
| BMI | 0.104 | 1 | 0.7467 | Satisfied |
| NYHA | 0.156 | 1 | 0.6931 | Satisfied |
| `Pulmonary arterial systolic pressure` | 0.415 | 1 | 0.5194 | Satisfied |
| lgBNP | 12.928 | 1 | 0.0003 | Violated |
| WBC | 0.526 | 1 | 0.4681 | Satisfied |
| Hb | 0.12 | 1 | 0.729 | Satisfied |
| Sodium | 7.091 | 1 | 0.0077 | Violated |
| Chlorine | 0.02 | 1 | 0.8865 | Satisfied |
| ALT | 0.025 | 1 | 0.8737 | Satisfied |
| AST | 1.388 | 1 | 0.2388 | Satisfied |
| Urea | 0.087 | 1 | 0.7679 | Satisfied |
| GFR | 7.283 | 1 | 0.0070 | Violated |
| FBG | 0 | 1 | 0.9892 | Satisfied |
| UA | 1.649 | 1 | 0.1991 | Satisfied |
| TC | 0.097 | 1 | 0.756 | Satisfied |
| Group | 0.05 | 1 | 0.8223 | Satisfied |
| GLOBAL | 44.705 | 18 | 0.0005 | Violated |
| The proportional hazards assumption was tested using Schoenfeld residuals. A p value > 0.05 indicates that the proportional hazards assumption is not violated. Global test represents the overall test for all covariates simultaneously. | | | | |
